# Family environment and development in children adopted from institutionalized care

**DOI:** 10.1038/s41390-020-01325-1

**Published:** 2021-05-26

**Authors:** Margaret F. Keil, Adela Leahu, Megan Rescigno, Jennifer Myles, Constantine A. Stratakis

**Affiliations:** 1 Section on Endocrinology and Genetics, Eunice Kennedy Shriver National Institute of Child Health and Human Development, National Institutes of Health, Bethesda, MD USA; 2 University of Nevada School of Medicine, Reno, NV USA; 3 Nutrition Department, Clinical Center, National Institutes of Health, Bethesda, MD USA

## Abstract

**Background:**

After adoption, children exposed to institutionalized care show significant improvement, but incomplete recovery of growth and developmental milestones. There is a paucity of data regarding risk and protective factors in children adopted from institutionalized care. This prospective study followed children recently adopted from institutionalized care to investigate the relationship between family environment, executive function, and behavioral outcomes.

**Methods:**

Anthropometric measurements, physical examination, endocrine and bone age evaluations, neurocognitive testing, and behavioral questionnaires were evaluated over a 2-year period with children adopted from institutionalized care and non-adopted controls.

**Results:**

Adopted children had significant deficits in growth, cognitive, and developmental measurements compared to controls that improved; however, residual deficits remained. Family cohesiveness and expressiveness were protective influences, associated with less behavioral problems, while family conflict and greater emphasis on rules were associated with greater risk for executive dysfunction.

**Conclusions:**

Our data suggest that a cohesive and expressive family environment moderated the effect of pre-adoption adversity on cognitive and behavioral development in toddlers, while family conflict and greater emphasis on rules were associated with greater risk for executive dysfunction. Early assessment of child temperament and parenting context may serve to optimize the fit between parenting style, family environment, and the child’s development.

**Impact:**

- Children who experience institutionalized care are at increased risk for significant deficits in developmental, cognitive, and social functioning associated with a disruption in the development of the prefrontal cortex. Aspects of the family caregiving environment moderate the effect of early life social deprivation in children.
- Family cohesiveness and expressiveness were protective influences, while family conflict and greater emphasis on rules were associated with a greater risk for executive dysfunction problems.
- This study should be viewed as preliminary data to be referenced by larger studies investigating developmental and behavioral outcomes of children adopted from institutional care.

## Introduction

The science of early childhood development is clear about the importance of early experiences, caregiving environment, and environmental threats on biological, cognitive, and behavioral development. Young children exposed to institutionalized care, which often corresponds with social deprivation and low caregiving quality, have an increased risk for behavioral problems and psychopathology.^1–6^ Intervention studies of children who experienced institutionalized care and are later adopted or placed into foster care provide evidence that a more favorable caregiving environment may lead to improved outcomes in growth, health, and development, and an overall reduced risk for psychopathology^7–11^ and may reverse the negative effects of early deprivation on hypothalamic pituitary axis functioning and neurobehavioral development.^8,12–20^

Prior studies have addressed the effects of institutionalized care on neurodevelopment and identified significant deficits in cognitive and social functioning, and developmental delay in children adopted post institutionalization.^3,5,6,8,21–28^ Age at adoption and time spent in institutionalization are associated with significant and often detrimental effects on overall outcomes.^21,22^ Institutionalized care and accompanying stimulus deprivation affect the development of the prefrontal cortex.^23,29–35^ The prefrontal cortex has a key role in the development and regulation of executive functions as well as the control of the autonomic system balance. Executive functions refer to a group of higher-order cognitive processes that coordinate the planning and execution of thoughts, emotions, and behaviors, as well as the storage of information in working memory.^36–39^ Executive skills are critical building blocks for the early development of cognitive and social capabilities; the gradual acquisition of these skills correspond to the development of the prefrontal cortex and other brain areas from infancy to adulthood.^36–39^

There is a paucity of research about post-adoption parenting styles that may promote recovery in children after institutionalized care. Ample evidence supports that the early caregiving environment is a consistent predictor of developmental outcomes and executive skills.^40–44^ The developing executive function system is influenced by a child’s experiences, response to stress, and structural and molecular changes associated with changes in the hormonal milieu in the brain during sensitive periods of development. Dehydroepiandrosterone (DHEA) has a critical role in human brain development and cognition likely due to the effects of this steroid in enhancing brain plasticity.^45,46^ Results of recent studies suggest that DHEA affects the development of cortico-amygdala^46^ and cortico-hippocampal functions^47^ that are important to encoding and processing of emotional, spatial, and social cues, as well as attention and working memory processes. In addition, steroids that are DHEA precursors, such as progesterone and allopregnanolone, have critical roles in neuroprotection.^36–39^

In this prospective study, we followed the development of children who experienced institutionalized care 2 years post adoption by a family in the United States. We examined the relationship between family environment, growth, endocrine and levels of neurosteroids, executive functioning, and cognitive development in children adopted from institutionalized care and non-adopted controls to identify factors related to developmental recovery and behavioral outcomes.

## Methods

### Participants

We recruited children adopted from institutionalized care in Eastern Europe within 2 months of adoption by a US family. Eligible participants had no history of significant medical, developmental, or behavioral problems. Participants were screened to determine that they spent at least 8 months in the institution/orphanage setting and were placed in the institution/orphanage at 6 months of age or less. Participants were recruited from local adoption referral centers. Child participants were  recruited for a control group and were cohort age–sex-matched with the adopted subjects. The controls were healthy children with no history of significant medical, psychological, or behavioral disorders. Exclusion criteria for the study included documented history of growth hormone deficiency, history of chronic illness (i.e., renal failure, chronic lung disease, diabetes, hypothyroidism, chromosomal abnormalities, medical conditions known to be associated with developmental delay (i.e., fetal alcohol syndrome (subjects were screened using criteria developed by Hoyme et al.^48^)) chronic infectious disease (e.g., AIDS, hepatitis), or precocious puberty. Socio-economic scores were similar between groups.

Participants were seen at baseline (within 2 months of arrival in the United States for adopted subjects) at 1- and 2-year follow-up. All studies were conducted under protocol 06-CH-0223 that was approved by the Eunice Kennedy Shriver National Institute of Child Health and Human Development Institutional Review Board. Informed consent was obtained from the parent/legal guardian. A total of 11 adopted children and 27 controls were recruited. Ten adopted children and 19 controls completed at least two follow-up visits and were included in the analysis. The study was closed to recruitment earlier than anticipated due to the suspension of adoptions from Eastern Europe to the United States.

### Measures

Anthropometric measurements, physical examination, neurocognitive testing, behavioral questionnaires, and endocrine labs and bone age (adopted children only) were evaluated over a 2-year period. Anthropometric measures included height, weight, body mass index (BMI), mid-arm circumference (MAC), triceps skinfold (TSF), subscapular skinfold (SSF), waist circumference (WC), and occipitofrontal circumference (OFC) by a registered dietitian.

Due to the participants’ age and ethical issues related to procedures that expose healthy child participants to risk, blood and bone age x-rays to assess nutritional and endocrine status were obtained for adopted children only (along with clinically indicated laboratory tests). Serum cortisol, DHEA, testosterone, estradiol, and serum neurosteroid profile were also collected (convenience sample: between 11 a.m. and 1 p.m.).

Neurocognitive testing was performed by a pediatric neuropsychologist and included either the Bayley III or Differential Abilities Scale II (DAS) based on age-appropriate guidelines. Behavioral questionnaires included Child Behavior Checklist (CBCL), Behavior Rating Inventory of Executive Function- Preschool (BRIEF-P), Infant Toddler Social Emotional Assessment (ITSEA), Colorado Child Temperament Inventory (CCTI), and Family Environment Scale (FES). Waters Attachment Behavior Q-sort (AQS) assessment of child attachment (Waters, SUNY) was performed by two trained observers at the initial visit.

The Bayley III is a clinical evaluation by a trained clinician to identify developmental issues in infants and toddlers and consists of the following domains, adaptive behavior, cognition, language, motor skills, and social–emotional capacities. Mean scores for scales are 10, with an SD of three.^49^ The DAS is a nationally normed (US) battery of cognitive and achievement tests for children aged 2 years 6 months to 17 years 11 months across a range of developmental levels; mean is 100, SD of 15.^50^ The CBCL questionnaire is a validated parent-report measure to assess emotional (internalizing and externalizing symptoms) and maladaptive behavior in children.^27^ The BRIEF-P is a reliable, valid parent-report inventory to assess executive function in preschool children; our analysis focused on the clinical scales of: inhibit (control behavioral response), shift (ability to alternate attention), emotional control (regulate emotional responses), working memory (ability to hold information when completing a task), plan/organization (to plan, organize), and Global Executive Composite (GEC). Scores on the CBCL and BRIEF-P are normalized to a mean of 50 (SD 10), with higher scores indicative of greater degrees of dysfunction and scores >65 considered to be clinically significant.^51^ ITSEA is a validated measure completed by the parent to assess social–emotional problems and competence in children (1–3 years of age) and is comprised of four domains, externalizing (impulsive, aggression), internalizing (depression, anxiety, separation distress, inhibition to novelty), dysregulation (sleep problems, negative emotions, sensory sensitivity), and competence (attention, compliance, play, mastery, empathy, prosocial peer relations).^52^

The CCTI is a validated inventory designed to assess the temperament of children by parental report.^53^ The FES is a self-reported questionnaire to assess social climate and environmental family characteristics and family functioning and emotions. The FES is categorized into three domains with ten subscales—relationship dimensions (cohesion, expressiveness, and conflict), personal growth dimensions (independence, achievement orientation, intellectual–cultural orientation, active–recreational orientation, and moral–religious aspect), and system maintenance dimensions (organization and control).^54^ The AQS is widely used to assess child attachment behavior and is based on Ainsworth’s study of secure attachment behavior in infants. The AQS assesses the correlation between secure attachment type and child–parent boundaries and has high validity. The AQS security score is the correlation of a specific child’s Q-sort to prototypical secure child and the score range is from −1.0 to +1.0.^55,56^

We hypothesized that aspects of the family environment, as measured by FES, would be associated with outcome measures of cognitive, executive function, and behavioral problems.

### Statistical analysis

To compare children of different ages, anthropometric measurements, and cognitive function scores were converted to *z*-scores (the difference between the child’s measurement/score and the age mean or the mean provided by standardized cognitive test, divided by the standard deviation (SD)). For length, height, weight, BMI, OFC, MAC, TSF, SSF, and WC *z*-scores were calculated using the program PediTools,^57^ based on means for age and SDs obtained by the National Health and Nutrition Examination Survey (Center for Disease Control and Prevention (CDC)). The CDC provides a set of growth measurements that are standardized among an ethnically diverse population.

Descriptive statistics were examined, and analysis of variance (ANOVA) was conducted to evaluate group differences in growth, cognitive, and behavior problems. Statistical comparisons included paired *t* tests, ANOVAs, correlation, and regression analysis. Regression analyses were conducted to examine which aspects of the family environment predicted cognitive or behavioral outcome measures. Analyses were conducted using the SPSS software. A *p* value <0.05 was considered for statistical significance.

## Results

### Subjects

There was no significant age or sex difference between adopted and control groups at the initial visit (adopted: 27.5 ± 9.3 months (range 14–40 months), 6 females, 4 males; control: 30.7 ± 14 months (range 10–58 months), 9 females, 10 males). For adopted subjects, the average time spent in institutionalized care was 23.6 ± 9 months. All the adopted children in our study were engaged with early intervention educational services.

### Growth

At baseline, adopted subjects had significantly lower *z*-scores for height/length, weight, OFC, and MAC compared to controls (*p* < 0.5). At baseline, one adopted subject had height and weight *z*-score <2 SD, compared to one subject in the control group with weight <2 SD; six adopted subjects had OFC <2 SD compared to one control subject with OFC <2 SD. No significant differences were found for *z*-scores for TSF or SSF or WC. At 2-year follow-up, adopted subjects showed significant improvement in *z*-scores of height and weight; there were no differences between the two groups for anthropometric measures. For adopted subjects at follow-up, one child had weight SD < 2 SD and four children had OFC < 2 SD. OFC was not obtained in most control subjects at 2-year follow-up. (Table 1).

**Table 1:** Clinical characteristics of study participants.

	Baseline			2-year Follow-up		
	Adopted, M (SD)	Control, M (SD)	*P* value	Adopted, M (SD)	Control, M (SD)	*P* value
Age (months)	27.5 (9.3)	30.7 (14)	n.s.	51.0 (10)	55.0 (14)	n.s.
Height/length	−1.24 (0.7)^a^	0.07 (1.1)	0.002*	−0.20 (1.0)	0.32 (1.1)	0.12
Weight	−1.35 (1.4)^a^	0.30 (1.0)	0.002*	−0.43 (1.4)	0.35 (0.9)	0.11
BMI	−0.33 (1.5)	0.52 (1.0)	0.05	−0.34 (1.3)	0.35 (0.7)	0.10
OFC	−1.88 (0.8)	0.21 (1.4)	<0.001*	−1.18 (1.3)^b^	–	–
MAC	−1.44 (1.5)	−0.43 (0.9)	0.034*	−1.32 (1.5)	−0.86 (0.9)	0.37
TSF	−1.22 (1.5)	−0.40 (0.7)	0.13	−0.92 (1.2)	−0.12 (0.9)	0.09
SSF	−0.64 (0.9)	−0.10 (0.7)	0.19	−0.76 (0.7)	−0.31 (0.6)	0.12
WC	−0.39 (0.4)	0.34 (0.8)	0.10	−0.31 (1.0)	−0.04 (0.7)	0.31
IGF-1	0.62 (0.2)	–	–	0.43 (0.3)	–	–
IGFBP3	1.2 (0.3)	–	–	1.58 (0.3)	–	–

*M* mean, *SD* standard deviation, *n.s.* not significant.

*Significant difference with *p* value < 0.05.

– Data not collected.

^a^Significant difference between baseline and 2-year follow-up.

^b^Three patients did not have OFC measured at 2-year visit, so 1-year data used.

Serum was obtained at baseline and 2-year follow-up. IGF-1 was quantified by chemiluminescence immunoassay performed by NIH Department of Laboratory Medicine, IGFBP3 was performed by enzyme-labeled chemiluminescent immunometric assay and performed by Mayo Laboratories. IGF-1 and IGFBP3 were reported as SD scores.

### Endocrine and metabolic measures (adopted children)

**Fig. 1:**
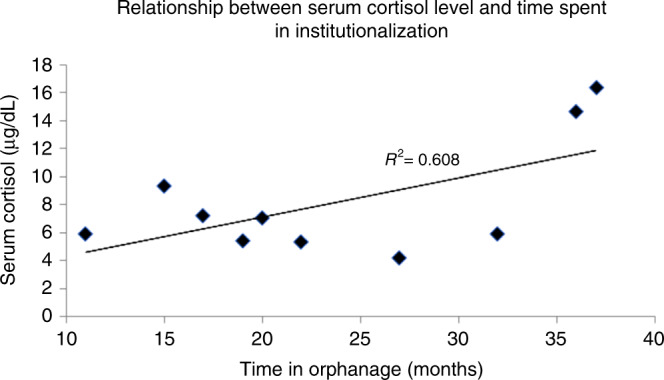
Cortisol. Cortisol levels in adopted children: time in orphanage care is positively correlated with serum cortisol at baseline (*r*^2^ = 0.608, *p* < 0.06). Serum cortisol was obtained between 11 am and 1 pm. (convenience sample). Cortisol levels ranged from 4.2 to 16.3 μg/dL.

Serum cortisol was obtained between 11 a.m. and 1 p.m. The range of cortisol levels was 4.2 to 16.3 μg/dL. Time in orphanage care was positively associated with serum cortisol at baseline (*R*^2^ = 0.61, *p* < 0.06) (Fig. 1). Due to the small sample size, the two outliers with longer time in orphanage care may have skewed the results; however, serum cortisol levels at follow-up were not statistically different from baseline values. We planned to collect salivary cortisol levels (diurnal) for both adopted and control subjects; however, due to poor compliance or lack of ample quantity of sample collected, there was insufficient data for analysis. At baseline, thyroid function results were within normal limits, except for one child who had mildly elevated thyroid-stimulating hormone with normal free T4, which normalized at follow-up visit. Other endocrine hormone levels were within normal limits for age/sex. Insulin-like growth factor-1 (IGF-1) and insulin-like growth factor-binding protein 3 (IGFBP3) *z*-scores at baseline (0.62 ± 0.2, 1.2 ± 0.3, respectively) and follow-up (0.43 ± 0.3, 1.58 ± 0.3, respectively) were within normal range. Growth factors were not a predictor of cognitive outcome. At the initial visit, bone age was consistent with chronological age in five children, advanced in three children, and delayed in two children. At follow-up, bone age was consistent with chronological age in six, advanced in two, and delayed in two children.

A serum lipid panel was obtained (convenience sample, non-fasting). At baseline, serum cholesterol and low-density lipoprotein levels were within normal limits for age. Serum high-density lipoprotein levels were <40 mg/dL in six of the ten subjects, and at follow-up remained <40 mg/dL in two of the nine subjects.

Serum neurosteroids were measured at baseline (*n* = 6) and follow-up (*n* = 9) by isotope dilution high-performance liquid chromatography-tandem mass spectrometry.^58^ Allopregnanolone levels were within the expected range for the assay and levels were similar to a recent report in a healthy population of toddlers that found no significant diurnal variation, as well as no differences between males and females, in the first 3 years of life.^59^ Serum tetrahydro-11 deoxycortisol, tetrahydrodeoxycorticosterone, and DHEA levels were at the lower limit of detection for the assay and did not change in the six subjects who had both baseline and follow-up measured (Table 2).

**Table 2:** Comparison of lipids and allopregnanolone in adopted children.

Patient	Baseline				Follow-up			
	Allopregnanolone (ng/mL)	Chol (mg/dL)	HDL (mg/dL)	LDL (mg/dL)	Allopregnanolone (ng/mL)	Chol (mg/dL)	HDL (mg/dL)	LDL (mg/dL)
1	–	113	33	81	0.0780	104	30	32
2	–	153	49	91	0.0480	164	46	70
3	–	124	49	57	0.0840	131	51	57
4	–	147	37	89	–	160	44	91
5	0.0845	136	27	96	0.0777	132	35	87
6	0.0565	146	40	72	0.0388	140	41	36
7	0.0596	125	37	61	0.0332	145	42	69
8	0.0499	125	43	68	0.0762	141	66	57
9	0.0518	150	34	–	0.0777	–	–	–
10	0.1130	150	46	87	0.0413	129	46	72
Reference range	0.01–0.72	<170	>35	<110	0.01–0.72	<170	>35	<110

– Data not collected.

### Cognitive data

**Fig. 2:**
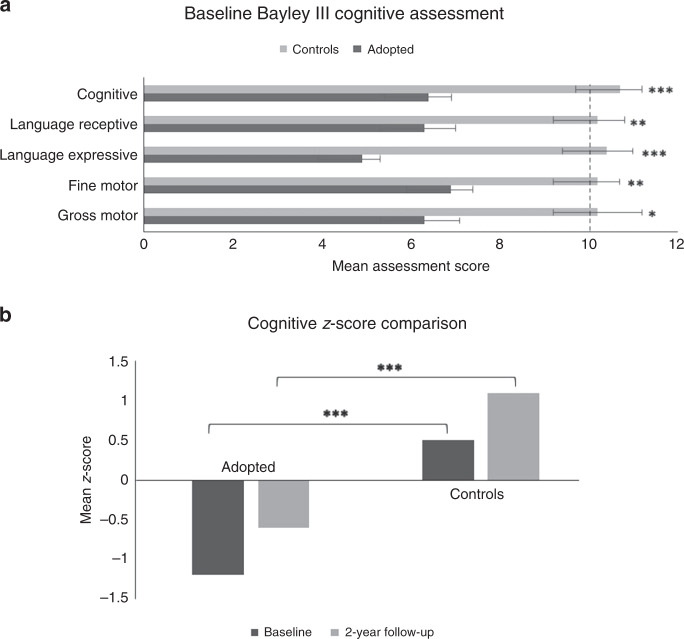
Comparison of cognitive scores at baseline and follow-up. **a** Comparison of mean scores on Bayley III at baseline. Adopted subjects had significantly lower scores in all subscales compared to controls. **b** Comparison of baseline and follow-up cognitive *z*-scores. Adopted subjects had significantly lower *z*-scores at baseline and although a trend was noted for improvement in adopted subjects’ scores from baseline to follow-up, residual differences remained. Error bars indicate standard error. *P < 0.05.

At baseline, adopted subjects had significantly lower scores compared to controls on all cognitive measures (Bayley III): cognitive, language receptive, language expressive, fine motor, and gross motor (*n* = 9 of adopted and 10 of controls were age appropriate for testing with Bayley III). To compare changes in scores from baseline to follow-up, overall cognitive *z*-scores were calculated (*z*-score of Bayley III or DAS General Cognitive Ability) and ANOVA analysis was performed. At baseline, general cognitive *z*-scores were significantly lower for adopted vs. controls; at 2-year follow-up, there was a trend for improvement in scores for adopted; however, residual differences remained compared to controls. For adopted subjects, lower OFC *z*-scores (baseline) were associated with lower cognitive scores at follow-up (Table 3 and Fig. 2).

**Table 3:** Behavioral index comparison.

	Baseline^a^			Follow-up^b^		
	Adopted, M (SEM)	Control, M (SEM)	*P* value	Adopted, M (SEM)	Control, M (SEM)	*P* value
BRIEF	*n* = 8	*n* = 19		*n* = 8	*n* = 14	
Inhibition	55 (7)	44 (2)	0.05^c^	59 (5)	47 (3)	0.04^c^
Shift	47 (3)	45 (2)	n.s.	50 (4)	44 (2)	n.s.
Emotional Control	42 (2)	42 (2)	n.s.	55 (3)	47 (3)	n.s.
Working Memory	44 (3)	44 (3)	n.s.	57 (6)	47 (3)	0.07
Plan/Organization	43 (3)	43 (3)	n.s.	53 (4)	45 (2)	n.s.
Inhibitory Self-control	42 (2)	42 (2)	n.s.	58 (4)	46 (3)	0.03^c^
Behavioral Flexibility	41 (2)	41 (2)	n.s.	53 (4)	45 (2)	n.s.
Metacognition	43 (3)	43 (3)	n.s.	54 (6)	46 (3)	n.s.
Global Executive Composite	42 (2)	42 (2)	n.s.	57 (5)	46 (3)	0.06
ITSEA	*n* = 9	*n* = 19				
Externalizing	46 (3)	45 (2)	n.s.	–	–	–
Internalizing	44 (3)	40 (2)	n.s.	–	–	–
Dysregulation	41 (4)	42 (3)	n.s.	–	–	–
Competence	31 (5)	51 (2)	0.001^c^	–	–	–
CCTI	*n* = 10	*n* = 19				
Social	18.5 (1)	13.3 (2)	n.s.	–	–	–
Emotional	12.3 (1)	8.0 (1)	0.03	–	–	–
Activity	18.0 (1)	12.5 (2)	n.s.	–	–	–
Attention	15.8 (1)	12.0 (2)	n.s.	–	–	–
CBCL	*n* = 9	*n* = 18		*n* = 8	*n* = 17	
Internalizing	44.6 (3)	38.4 (2)	n.s.	51.0 (4)	43.0 (2)	0.08
Externalizing	46.0 (4)	41.3 (3)	n.s.	53.0 (6)	40.0 (2)	0.03^c^
Total Problems	33.6 (3)	33.0 (1)	n.s.	37.5 (2)	33.0 (1)	0.09

*M* mean, *SEM* standard error of the mean, *n.s.* not significant.

^a^Completed by parent within 2 months of arrival in United States for adopted subjects.

^b^Completed by parent at 2-year follow-up.

^c^Significant difference with *p* < 0.05.

– data not collected.

### Behavioral data

At baseline, adopted children had significantly lower scores than controls for the ITSEA competence subscale (*p* < 0.001; *F* = 19.017); lower scores are associated with lower social–emotional competence. Since most subjects were above the age limit for use of ITSEA at follow-up, these data were not included in the analysis. At baseline, adopted children had significantly higher scores on the emotional subscale of the CCTI compared to controls (*p* < 0.03; *F* = 5.516). Baseline CBCL results showed no difference between the adopted and control group for any subscale scores. At 2-year follow-up, adopted children had significantly higher scores on externalizing symptom subscales compared to controls (*p* < 0.03; *F* = 5.251).

**Fig. 3:**
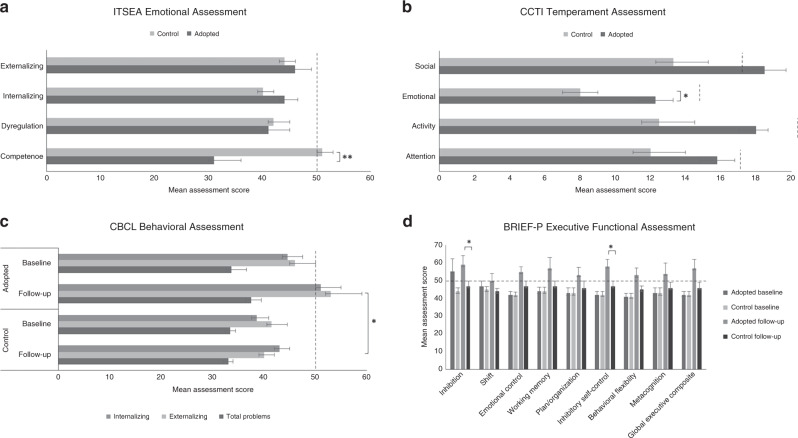
Comparison of behavioral, emotional, temperament, and executive function scores. Comparison of mean scores on **a** ITSEA-Emotional Assessment (baseline); **b** CCTI-Temperament Assessment (baseline); **c** CBCL-Behavioral Assessment (baseline and follow-up); and **d** BRIEF-P-Executive Function (baseline and follow-up) of adopted vs. controls. Error bars indicate standard error. *P < 0.05.

For adopted subjects at baseline, parent responses for the BRIEF endorsed clinically significant inhibitory control in half the children (*p* < 0.05; *F* = 4.424); no significant difference was found between the adopted and control groups for other subscales. At follow-up the adopted group had significantly higher scores (higher scores associated with more problems) compared to controls for the following subscales: inhibition (*p* < 0.04; *F* = 5.027), inhibitory self-control (*p* < 0.03; *F* = 5.328), with a trend noted for working memory and GEC (Fig. 3).

Waters Q attachment scores showed no difference in attachment between adopted children and controls; AQS scores strongly correlated with norms for a sensitive response. Based on that, we concluded that there were no differences between parents’ sensitivity and child attachment in either group and their secure–insecure attachment distribution was comparable with that of normative groups (data not shown). FES scores at baseline showed a significant difference for only the independence subscale score between adopted vs. control groups (*p* < 0.05; *F* = 4.418).

To identify sociodemographic and family environment factors associated with increased risk for executive dysfunction or behavioral problems, a correlational analysis was performed between demographic variables of child gender and age and executive function variables to determine possible covariate variables. Sex was not significantly correlated with any executive function variables and therefore not included in any future analysis. However, age at baseline was significantly correlated with BRIEF subscales; correlation and linear regression analyses were used for these executive function variables.

For adopted subjects, the baseline FES subscales control and conflict were predictors of higher GEC scores at follow-up (BRIEF measure; higher scores associated with dysfunction) (*R*^2^ = 0.91; *F* = 14.48, *p* = 0.03). FES subscale achievement positively correlated with change in cognitive *z*-scores (*R*^2^ = 0.433; *F* = 6.106, *p* = 0.04). FES subscales cohesion and expressiveness were negatively associated with a change in internalizing scores of CBCL (*R*^2^ = −0.9; *p* = 0.04), that is, greater cohesion and expressiveness were associated with lower scores on internalizing symptoms of CBCL. FES subscale control was a predictor of a higher internalizing score (CBCL) at follow-up (*R*^2^ = 0.74; *F* = 10.893, *p* = 0.03); greater emphasis on rules and procedures were associated with more internalizing symptoms, which is a reflection of mood disturbance (i.e., anxiety, depression, social withdrawal). CCTI emotionality was associated with an increase in externalizing scores of CBCL for adopted subjects (*R*^2^ = 0.97; *p* < 0.005) (Tables 4 and 5).

**Table 4:** Family Environment Scale (Baseline).

	Adopted, M (SEM), *n* = 10	Control, M (SEM) *n* = 19	*P* value
Cohesion	61.0 (2)	56.0 (6)	n.s.
Expressiveness	55.3 (3)	58.0 (6)	n.s.
Conflict	42.3 (3)	38.0 (4)	n.s.
Independence	57.0 (3)	43.0 (4)	0.05
Achievement	40.5 (3)	43.0 (4)	n.s.
Intellectual	56.6 (3)	57.0 (4)	n.s.
Active	52.5 (3)	49.0 (4)	n.s.
Moral	50.0 (3)	51.0 (4)	n.s.
Organizational	52.3 (3)	53.0 (4)	n.s.
Control	41.0 (3)	42.0 (4)	n.s.

*M* mean, *SEM* standard error of the mean, *n.s.* not significant.

**Table 5:** Pearson correlations of FES subscales and cognitive-behavioral outcomes for adopted subjects.

FES subscale	GEC follow-up	Delta CBCL internalizing	Delta cognitive *z*-score
Control	0.82 (*p* = 0.02)*	0.73 (*p* = 0.03)*	–
Conflict	0.90 (*p* = 0.01)*	–	–
Cohesiveness	–	−0.96 (*p* = 0.01)*	–
Expressiveness	–	−0.90 (*p* = 0.04)*	–
Achievement	–	–	0.55 (*p* = 0.05)*

– Results non-significant.

*Significant difference with *p* value < 0.05.

## Discussion

This prospective study followed the development of children adopted from institutionalized care for 2 years post adoption compared to controls. Broadly, our findings are consistent with the literature, showing significant but not complete growth and developmental recovery post adoption for children exposed to institutionalized care. Kroupina et al.^28^ reported that growth factors (IGFBP3) at baseline were a negative predictor and change of head circumference and cognitive scores at 6 months were positive predictors, of cognitive outcomes at 30 months post adoption. Our data did not show a correlation between baseline growth factor *z*-scores and cognitive outcome at follow-up, perhaps due to the constraints of our small sample size. However, OFC *z*-scores at baseline were a predictor of cognitive scores at 2-year follow-up. Also, Kroupina et al.^28^ reported that smaller stature at baseline and weight gain were associated with improved height outcome at 30- month follow-up, and younger age and lower weight at baseline were a predictor of better catch-up growth. Our data did not replicate the findings of Kroupina et al.^28^ regarding predictors of catch-up growth, likely due to the constraints of our sample size. Baseline *z*-scores for height, weight, and OFC were similar between our study and Kroupina et al.,^28^ which had a larger sample size. As expected, there was a negative correlation between time in orphanage care and baseline height and weight *z*-scores. Consistent with previous studies,^8,21,24,26,34,60–64^ our results support specific aspects of the family environment that are associated with executive function and behavioral symptomology 2 years after adoption.^65,66^ Specifically, greater conflict and less flexible rules in a family were predictors of higher scores of global executive dysfunction. BRIEF scores reflect the parent’s observations of the child’s everyday executive functioning relative to the parent’s expectations (not an absolute level of functioning) and thus serve as a screening tool for executive dysfunction. Also, in this study, adopted children were found to have higher scores for behavioral inhibition, an aspect of temperament characterized as social reticence that is reported to be stable across childhood and is associated with greater risk for developing social withdrawal, anxiety disorders, and internalizing problems. Prior studies report that developmental outcomes associated with behavioral inhibition can be influenced by the caregiving context; authoritarian style (i.e., lack of emotional warmth, non-transparent declaration of rules, and high levels of control) is detrimental for social developmental outcomes.^67^

Family cohesion and expressiveness were a protective influence; at 2-year follow-up, stronger family cohesion and expressiveness were associated with lower internalizing scores (i.e., less problems with mood disturbance, including anxiety, depression, and social withdrawal). Prior studies of internationally adopted children reported either higher mean internalizing symptoms or no differences in internalizing scores between adopted vs. non-adopted children.^66,68,69^ Consistent with prior studies, we found higher externalizing scores (i.e., greater problems with aggression, conflict, and violation of social norms) on the CBCL at 2-year follow-up for adopted children that were associated with higher emotionality scores on CCTI.^70^ Scores on the FES at baseline did not differ significantly between groups, suggesting that there were no differences in perceived family characteristics between adopted and controls.^54^

As expected, at baseline visit there were significant differences in measures of cognitive function between adopted children and controls; overall mean scores improved but remained lower than controls at 2-year follow-up. Cognitive scores were negatively associated with OFC *z*-scores (baseline visit). At baseline, compared to controls, adopted children scored lower on measures of competence (as measured by ITSEA) and scored higher (associated with more problems) on measures of emotionality (as measured by CCTI) and inhibitory control (as measured by BRIEF). At follow-up, adopted children scored higher (associated with more problems) on measures of externalizing symptoms, inhibition, inhibitory self-control, behavioral flexibility, working memory, and GEC (BRIEF). The developing executive function system is influenced by a child’s experiences and response to stress, which impacts the developing prefrontal cortex. In this study, although the measurement of neurosteroids did not reveal any relationship to measures of cognitive or behavioral symptomology; the small sample size and lack of data in the control group limit interpretation and future research is warranted.

We did not identify differences in attachment measures in adopted vs. controls. We observed “indiscriminate friendliness” in many of the adopted subjects, as has been described in the literature.^5,63^ Our observations are consistent with prior studies that note indiscriminate sociability in children with secure attachment.^71,72^

The strengths of this study are the prospective design and the differentiation of behavioral issues noted at adoption placement versus those that manifest later. Limitations of the study include the small number of participants (the study was terminated prematurely due to the cessation of adoptions from East Europe). Another limitation was that measures of internalizing, externalizing behaviors, and executive function included only parental assessments of behavior. Also, the lack of salivary cortisol data (due to either inadequate quantity of samples collected or poor compliance with collection in this infant/toddler population) is regrettable since salivary cortisol levels are widely used and are an invaluable tool for pediatric studies and would have provided useful information for comparison of adopted and control subjects.

This study, in the context of a small sample size, should be viewed as a pilot study in the field of developmental pediatrics. Here we find that specific aspects of the family caregiving environment moderate the effects of social deprivation during early childhood on executive function and behavioral problems. These findings provide preliminary data for larger studies that will further investigate the developmental effects that manifest in institutionalized children.

## Conclusion

In summary, findings from this study support a cohesive and expressive family environment moderated the effect of prior pre-adoption adversity on cognitive and behavioral development in toddlers. Family conflict and greater emphasis on rules/procedures were associated with a greater risk for behavioral problems at 2-year follow-up. Early assessment of child temperament child and parenting context may provide useful information to optimize the fit between parenting style, family environment structure, and the child’s development.
